# INFORMAL SUPPORT NETWORKS AND LONELINESS IN OLDER WOMEN LIVING WITH HIV

**DOI:** 10.1093/geroni/igad104.0447

**Published:** 2023-12-21

**Authors:** Jasmine Manalel, Mark Brennan-Ing

**Affiliations:** Hunter College - CUNY, New York City, New York, United States; Hunter College - CUNY, New York City, New York, United States

## Abstract

Older adults with HIV are at increased risk for social isolation and poor well-being. Although older women living with HIV (WLWH) are more likely to be embedded in supportive networks than men, they often report inadequate support in meeting their needs. The function of older WLWH’s informal networks, their effectiveness in meeting care needs, and links to well-being are major gaps in the literature. Using a subsample of heterosexual WLWH from The Research on Older Adults with HIV 2.0 Study aged 50 to 77 (Mage = 59; n = 210), we assessed sources of social support, interpersonal strain, and support adequacy, in relation to loneliness. About half the sample reported having ever (i.e., currently or in the past) needed regular assistance due to HIV or other illness, disability, or frailty whereas the rest reported never needed regular assistance. Preliminary regression analyses controlled for age, race, ADLs, partnership, and formal care status. Findings revealed that neither support from family or from friends was associated with loneliness. However, strain from both sources was significantly associated with greater loneliness. In terms of support adequacy, having someone to count on for emotional support was associated with lower loneliness, and needing more emotional support was associated with greater loneliness. Instrumental support (i.e., help with tasks was not significantly associated with loneliness. These findings highlight the importance of considering positive and negative social relations, as well as different dimensions and sources of support. Interventions to improve emotional support resources could help mitigate loneliness among older WLWH.

